# Disentangling heterogeneity in contemporary undifferentiated arthritis – A large cohort study using latent class analysis

**DOI:** 10.1016/j.semarthrit.2023.152251

**Published:** 2023-08-09

**Authors:** N.K. den Hollander, M. Verstappen, B.T. van Dijk, A.H.M. van der Helm - van Mil, H.W. van Steenbergen

**Affiliations:** a Department of Rheumatology, Leiden University Medical Center, Leiden, Netherlands; b Department of Rheumatology, Erasmus Medical Center, Rotterdam, the Netherlands

**Keywords:** Rheumatoid arthritis, Undifferentiated arthritis, Subgroups, RA-development, spontaneous resolution

## Abstract

**Objectives:**

Undifferentiated arthritis(UA) is clinically heterogeneous and differs in outcomes ranging from spontaneous resolution to RA-development. Therefore, we hypothesized that subgroups exist within UA and we aimed to identify homogeneous groups based on clinical features, and thereafter to relate these groups to the outcomes spontaneous resolution and RA-development. These outcomes can only be studied in UA-patients in which DMARD-treatment does not influence the natural disease course; these cohorts are scarce.

**Methods:**

We studied autoantibody-negative UA-patients (not fulfilling 1987/2010 RA-criteria, no alternate diagnosis), included in the Leiden Early Arthritis Clinic between 1993 and 2006, when early DMARD-treatment in UA was infrequent. Latent class analysis was used to identify subgroups based on combinations of clinical features. Within these subgroups, test-characteristics were assessed for spontaneous resolution of arthritis and RA-development within 1 year.

**Results:**

310 consecutive UA-patients were studied. Five classes were identified: location and number of swollen joints were most distinguishing. Classes were characterized by: 1) polyarthritis, often symmetric; 2) oligoarthritis, frequently with subacute onset; 3) wrist-monoarthritis, often with subacute onset, increased BMI and without morning stiffness; 4) small-joint monoarthritis, often without increased acute phase reactants, and 5) large-joint monoarthritis, often with subacute onset. Studying the classes in relation to the outcomes revealed that patients without spontaneous resolution (thus having persistent disease) were nearly absent in the classes characterized by monoarthritis (specificity >90%). Additionally, patients who developed RA were infrequent in monoarthritis classes (sensitivity <7%).

**Conclusion:**

Using a data-driven unsupervised approach, five subgroups within contemporary UA were identified. These have differences in the natural course of disease.

## Introduction

Undifferentiated arthritis (UA) is a clinically heterogenous disease, as illustrated by the variable outcomes. UA-patients can either progress to rheumatoid arthritis (RA), remain ‘undifferentiated’ or achieve spontaneous resolution of arthritis without DMARD-treatment [1]. This variability in outcomes might indicate that subgroups are present within the UA-population.

In the last decades, the definition and treatment strategies of UA changed. Since the introduction of the 2010 RA-criteria, autoantibody-positive patients who were formerly considered as UA are now classified as RA earlier in the disease course. The contemporary UA-population is defined as neither fulling 1987 nor 2010 RA-criteria. The contemporary UA-population was recently described as mostly autoantibody-negative and patients generally have few swollen joints [1,2]. However, the UA-population according to this definition has scarcely been studied. In addition to a change in the UA-population during the last decade, treatment strategies in UA have changed: DMARD-treatment is nowadays initiated early in UA, as recommended in EULAR-guidelines [2–4]. This tendency to start DMARD-treatment early hampers evaluating the natural course of patients nowadays presenting with contemporary UA [1].

In the present study we took advantage of unique data on consecutive UA-patients in an era in which DMARD-treatment of UA was still infrequent and disease outcomes were marginally influenced by early DMARD-treatment.

A statistical method that specifically aims to group individuals with similar characteristics is latent class analysis (LCA). This is a “person-centred approach” and in contrast to regression analyses, which focusses on relationships amongst variables with the intention to predict outcomes, LCA does not include outcomes [5,6].

We aimed to distinguish subgroups within contemporary UA-patients, based on a combination of clinical characteristics at first presentation, using LCA. The identified subgroups were also studied in relation to two outcomes: RA-development and spontaneous resolution.

## Patients and methods

### Patients

Patients with contemporary UA, consecutively included in the Leiden EAC (supplementary data 1) between February 1993 and January 2006 and who were autoantibody-negative were selected [7]. The Leiden Early Arthritis Clinic (EAC) is a population-based inception-cohort, described in detail elsewhere [7]. In short, since 1993 patients presenting with recent-onset arthritis of ≥1 joint and symptom duration <2 years have been consecutively included. At regular research visits (baseline, 4 months and yearly thereafter) clinical characteristics were assessed, joint counts were performed and IgM-RF (in-house ELISA, considered elevated if ≥5.0 IU/ml), CRP and ESR measured. ACPA was assessed retrospectively, using Euro-diagnostics assay in preserved baseline samples.

UA was retrospectively defined as clinical arthritis of ≥1 joint without fulfilling 1987/2010 RA-classification criteria and having no alternate clinical diagnosis according to the rheumatologist [8,9]. Both 1987 and 2010 RA-classification criteria were incorporated in the definition of contemporary UA, because the 2010-criteria identify autoantibody-positive RA-patients earlier, while the 1987-criteria are more accurate in early identification of autoantibody-negative RA [10, 11]. Imaging findings were not included in defining joint involvement for the 2010 criteria [12].

The period 1993–2006 was chosen since early DMARD-treatment in UA was still infrequent in that era, limiting the influence of DMARD-treatment on the outcomes [1,3]. Only autoantibody-negative patients were studied, as autoantibody-positive patients are often considered RA-patients by rheumatologists, also without fulfilling RA-criteria.

### Outcomes

Two outcomes were retrospectively assessed at 1 year of follow-up: 1) RA-development defined as fulfilment of 1987/2010 RA-criteria and 2) spontaneous resolution of arthritis. Spontaneous resolution was defined as sustained absence of clinical arthritis without DMARD-use within 1 year and no clinical arthritis or DMARD use during the entire follow-up thereafter.

### Variables of interest

Variables of interest were characteristics that are generally available or collected during a first consultation at the rheumatology outpatient clinic. These were: patient demographics (age and sex), presence of relatives with RA, symptom characteristics (subacute/gradual onset, presence of morning stiffness ≥60 min), findings of physical examination (number and location of swollen joints; symmetry of swollen joints) and acute phase reactants (ESR and CRP). Subacute onset was defined as onset of symptoms within one week. Number of swollen joints was categorized into mono-, oligo-(2–4 joints) and polyarthritis(>4 joints). Symmetry of swollen joints was defined as symmetric involvement of the same joint areas. ESR was considered elevated depending on age and gender (<50 years: Male >15 mm/h, Female >20 mm/h; >50 years: male >20 mm/h, female>30 mm/h considered elevated). CRP was considered elevated if CRP ≥10 mg/L.

### Statistical analyses

Latent class analysis (LCA) was used to find subgroups based on combinations of clinical features at baseline. LCA searches for groups of patients (classes) with similarities in (combinations of) features and is a person-centred approach [5,6]. LCA increases the number of classes, until the best model is identified. Next to clinical relevance, multiple statistical measures are used to evaluate the best fit model and compare the model with the newer model with the previous model with one class less [6,13]. The improvement in fit between the current model and the model with one class less was compared using the Lo-Mendell-Rubin adjusted likelihood ratio test. The adjusted Bayesian Information Criterion (aBIC), Bayesian Information Criterion (BIC) and Akaike’s Information Criterion (AIC) were also used to select the best fit model. A lower Information Criterion indicates a better model fit. Lastly the entropy was assessed, which indicates how accurately the model defines the classes. Entropy ranges from 0 to 1 and a higher value indicates a more precise assignment of an individual patient in that specific latent class. An additional explanation regarding LCA can be found in supplementary data 1.

After identifying the best fit model, the LCA then yields 1) a probability of class-membership and 2) the probability of the observed variable(clinical characteristic) within and across the classes. Consequently, the percentage of patients within each class or percentage of patients with a certain clinical characteristic cannot be determined based on LCA alone and was assessed separately. Based on these probabilities, each class was labelled by its most recognizable feature(s).

We studied for each class the frequency of the long-term outcomes and determined test-characteristics. IBM SPSS Statistics v25, Statcorp Stata v.16 and Muthen&Muthen Mplus v7 were used. Ethics committee approval was received from ‘Commisie Medische Ethiek’ of the Leiden University Medical Centre (B19.008).

## Results

### Study population

Of 1769 early arthritis patients, 450 had contemporary UA. 310 patients who were autoantibody-negative and had complete outcome data (supplementary figure 1) were studied. These patients had similar characteristics as patients with missing data (supplementary Table 1). Mean age was 49 years, 52% were female patients and median SJC was 2 (Table 1).

### Subgroups in contemporary UA based on latent class analysis

A 5-class model best fitted the data (supplementary Table 2). Classes mainly differentiated regarding number and location of swollen joints (Table 2). Class 1 was characterized by polyarthritis, which was mainly symmetric. Patients in class 2 had oligoarthritis and often a subacute onset of symptoms. Patients with monoarthritis of the wrist were in class 3, often with a BMI>25, a subacute onset and absence of morning stiffness. Class 4 was characterized by small joint monoarthritis (39% probability for MCP1–5/MTP2–5 and 39% PIP/DIP 1–5, the remaining 22% of the patients had monoarthritis of MTP1 and the first interphalangeal joint of the foot) furthermore, patients were often younger than 50 years, predominantly had a BMI<25 and acute phase reactants were frequently normal. Finally, class 5 was characterized by large joint monoarthritis and had most often a subacute disease onset. For reasons of simplicity, classes were labelled according to their main feature as respectively ‘polyarthritis’, ‘oligoarthritis’, ‘monoarthritis wrist’, ‘monoarthritis other small joint’ and ‘monoarthritis large joint’. Baseline characteristics per class are shown in supplementary Table 3.

### Identified classes in relation to RA-development and spontaneous resolution

Of all UA-patients, 14% developed RA at 1 year after inclusion. For classes 1 to 5, this was respectively 21%, 19%, 7%, 10% and 2%. Test-characteristics were determined for the subgroups (Table 3). Notably, the three monoarthritis classes were related to less RA-development. Especially the sensitivity for RA-development was low in the mono-arthritis classes: 5%, 7% and 2% for monoarthritis of the wrist, small-joints and large joints respectively. Thus, patients who developed RA were almost absent in monoarthritis classes (<7%). Oligo-and poly-arthritis classes had less distinct sensitivities and specificities for RA-development.

Regarding spontaneous resolution, 49% of all UA-patients achieved spontaneous resolution during the first year. These patients remained in spontaneous resolution without receiving DMARD-treatment during their entire follow-up of median 20 years (IQR 11–24). In total 21% of all patients received DMARD-treatment in the first year of follow-up, these patients could per definition not achieve spontaneous resolution. For classes 1 to 5, respectively 34%, 42%, 59%, 55% and 72% achieved spontaneous resolution. Especially specificity was high in the three monoarthritis classes (>90%, Table 3). Thus patients without spontaneous resolution were almost absent in the monoarthritis classes (<10%). Oligo- and polyarthritis classes had less apparent sensitivities and specificities for spontaneous resolution.

## Discussion

This study identified five clinically recognizable subgroups within contemporary UA-patients, using a data-driven LCA. These subgroups are mainly based on number and location of swollen joints. Other features like disease onset, increased acute phase reactants, morning stiffness and BMI also contributed. To our knowledge this is the first study identifying subgroups in contemporary-UA based on clinical characteristics known at presentation, using an unsupervised person-orientated method.

The robustness of these subgroups was supported by differences in long-term outcomes (RA-development and spontaneous resolution of arthritis) between the subgroups. Interestingly, although outcomes were not included as feature of interest in the LCA, differences in these outcomes between the subgroups were found. Patients with unfavourable outcomes(RA-development and no spontaneous resolution) were hardly included in the three monoarthritis subgroups. Notably, the LCA identified three distinct monoarthritis subgroups. Besides differences in localization of arthritis, other features such as subacute onset, increased BMI, morning stiffness and acute phase reactants varied. Long-term outcomes also slightly differed for these three monoarthritis subgroups.

The variables included in the LCA had limitations and benefits. LCA can be used as an exploratory statistical approach, but ideally the choice of included variables should be guided by theory [6]. In this study, we selected variables that are always available, generally collected at first rheumatologic consultation and important for decision-making in clinical practice (e.g. number of involved joints). This helps with the interpretation of results and relating the results more easily to clinical practice. It is unknown if the subgroups identified by the LCA would have been different if additional variables or other (unavailable) clinical variables (i.e. inflammatory back pain, uveitis), genetics and imaging were included.

A strength of LCA is that it is a “person-centred approach”: it focusses on identifying patients with similar characteristics, instead of relationships between variables like linear regression. Another advantage of LCA is that researchers can only influence the selection of clinical factors and study population, while the analysis cannot be influenced: the analysis determines the model and subgroups, without pre-assumptions.

We studied a unique set of consecutive UA-patients who were included and followed in an era in which DMARD-treatment of UA was infrequent. Consequently, the possible influence of DMARD-treatment on the disease course was limited and we were practically able to study the natural disease course [3]. In more recent cohorts, assessing spontaneous resolution in UA is not possible due to the increased tendency to initiate DMARD-treatment [14]. To our best knowledge, other cohorts of patients with untreated UA do not exist. This highlights the unique setting of our study, but also implies that validating our results is difficult.

Spontaneous resolution was defined as sustained absence of clinical arthritis without DMARD-use within 1 year and no clinical arthritis or DMARD use during the entire follow-up thereafter. Due to this stringent definition, patients with only a short period of (DMARD) treatment or patients with initial spontaneous resolution and a flare years later (during median follow-up of 20 years) do not fulfil our definition of spontaneous resolution. However, this rather stringent definition was deliberately chosen to ensure sustainability of the spontaneous resolution and to assess an outcome which was the opposite of the outcome RA-development.

Another strength is the long observational follow-up of median 20 years. By following patients for median 20 years, it is not likely that the patients whom were considered having spontaneous resolution, could have developed arthritis or used DMARDs thereafter. As such, this shows the robustness of the outcome spontaneous resolution: all patients who achieved spontaneous resolution during the first year remained in spontaneous resolution during their entire follow-up.

Our primary research question was identifying subgroups and not performing prediction. Therefore our findings cannot be used as predictors within individual patients. However, to our best knowledge this is the first study that substantiates the hypothesis that recognizable subgroups are present in contemporary UA.

Concluding, this unique data-driven cohort-study identified 5 sub-groups of contemporary UA-patients. These subgroups differed in outcomes: patients with RA-development and without spontaneous resolution were rarely included in the monoarthritis subgroups.

## Supplementary Material

Supplemental file

## Figures and Tables

**Table 1:** Baseline characteristics of the autoantibody negative study population (*n* = 310).

	All autoantibody negative UA-patients *n* = 310
Age at inclusion (years)	49.0 (17)
Male sex	148 (48%)
Morning stiffness ≥60min	69 (22%)
Symptom duration (days)	92 (31–222)
Subacute onset (<1 week)	195 (63%)
Relative with RA	46 (15%)
Swollen joint count (68-joints)	2[1–4]
Monoarthritis	115 (37%)
Oligoarthritis	113 (37%)
Polyarthritis	62 (20%)
Tender joint count (71-joints)	2(1–4)
Symmetry of swollen joints	92 (30%)
Elevated CRP	126 (41%)
Elevated ESR	130 (42%)

Data are n (%), mean (SD) or median (IQR). Onset of symptoms was considered subacute in case the symptom duration was <1 week. CRP was considered elevated if ≥10 mg/L, ESR was considered elevated depending on age and gender (<50 years: Male >15 mm/h, Female >20 mm/h; >50 years: male >20 mm/h, female>30 mm/h considered elevated).

**Table 2:** Best fit 5-class model with the conditional probability of each clinical feature per class.

	Class 1 ‘polyarthritis’ 0.20	Class 2 ‘oligoarthritis’ 0.43	Class 3 ‘monoarthritis wrist’ 0.09	Class 4 ‘monoarthritis other small joint’ 0.10	Class 5 ‘monoarthritis large joint’ 0.18
Age>50	0.51 (0.38; 0.63)	0.55 (0.46; 0.63)	0.56 (0.37; 0.75)	0.29 (0.13; 0.44)	0.41 (0.28; 0.53)
Male sex	0.40 (0.28; 0.52)	0.50 (0.41; 0.59)	0.52 (0.33; 0.71)	0.29 (0.13; 0.44)	0.60 (0.47; 0.73)
BMI>25	0.44 (0.25; 0.62)	0.47 (0.35; 0.59)	0.73 (0.46; 0.99)	0.20 (0.00; 0.39)	0.43 (0.25; 0.61)
Morning stiffness ≥60min	0.22 (0.11; 0.33)	0.31 (0.22; 0.40)	0.08 (0.00; 0.18)	0.22 (0.07; 0.37)	0.18 (0.08; 0.28)
Relative with RA	0.14 (0.05; 0.23)	0.13 (0.07; 0.19)	0.35 (0.16; 0.53)	0.17 (0.04; 0.31)	0.13 (0.04; 0.23)
Subacute onset	0.54 (0.41; 0.66)	0.66 (0.57; 0.74)	0.74 (0.57; 0.91)	0.61 (0.44; 0.78)	0.76 (0.65; 0.88)
Monoart MCP1-5/MTP2-5	0.00 (0.00; 0.00)	0.00 (0.00; 0.00)	0.00 (0.00; 0.00)	0.39 (0.23; 0.55)	0.00 (0.00; 0.00)
Monoart PIP/DIP	0.00 (0.00; 0.00)	0.00 (0.00; 0.00)	0.00 (0.00; 0.00)	0.39 (0.23; 0.55)	0.00 (0.00; 0.00)
Monoart wrist	0.00 (0.00; 0.00)	0.00 (0.00; 0.00)	1.00 (1.00; 1.00)	0.00 (0.00; 0.00)	0.00 (0.00; 0.00)
Monoart large joint	0.00 (0.00; 0.00)	0.00 (0.00; 0.00)	0.00 (0.00; 0.00)	0.00 (0.00; 0.00)	1.00 (1.00; 1.00)
Oligoarthritis	0.00 (0.00; 0.00)	1.00 (1.00; 1.00)	0.00 (0.00; 0.00)	0.00 (0.00; 0.00)	0.00 (0.00; 0.00)
Polyarthritis	1.00 (1.00; 1.00)	0.00 (0.00; 0.00)	0.00 (0.00; 0.00)	0.00 (0.00; 0.00)	0.00 (0.00; 0.00)
Symmetry of swollen joints	0.68 (0.56; 0.80)	0.39 (0.30; 0.48)	0.00 (0.00; 0.00)	0.00 (0.00; 0.00)	0.00 (0.00; 0.00)
Acute phase reactants elevated	0.56 (0.44; 0.69)	0.56 (0.47; 0.65)	0.48 (0.29; 0.67)	0.24 (0.10; 0.39)	0.56 (0.43; 0.69)

The probability (0-[1]) of each feature is presented per class. If the probability is 1.00, all patients in that class have that feature. Thus, within class 1 polyarthritis is the classifying feature. The author labelled **dark cyan** if they were dominant/had a high probability (≥0.70) across a class (i.e. differentiating between classes). Features were labelled **light cyan** if they had a low probability (≤0.30) across a class. Features were labelled **mid cyan** if they were an important predominant characteristic within a class, but not dominant (≥0.70) across classes. Onset of symptoms was considered subacute in case the symptom duration was <1 week. BMI, body mass index; Monoart, monoarthritis; MCP, metacarpophalangeal joint; MTP, metatarsophalangeal joint; PIP, proximal interphalangeal joint; DIP, distal interphalangeal joint.

**Table 3:** Test-characteristics presented for each class for the outcomes RA-development and spontaneous resolution at 1 year.

	RA within 1 year (n)	No RA within 1 year (n)	Sensitivity (95% CI)	Specificity (95% CI)
Class 1 – Polyarthritis (*n* = 62)				
Class 1	13	49	30%	82%
Not in class	31	217	(18%–44%)	(76%–86%)
1				
Class 2 – Oligoarthritis (*n* = 133)				
Class 2	25	108	57%	59%
Not in class	19	158	(42%–70%)	(53%–65%)
2				
Class 3 – Monoarthritis wrist (*n* = 27)				
Class 3	2	25	5%	91%
Not in class	42	241	(1%–15%)	(86%–94%)
3				
Class 4 – Monoarthritis small joint (*n* = 31)				
Class 4	3	28	7%	89%
Not in class	41	238	(2%–18%)	(85%–93%)
4				
Class 5 – Monoarthritis large joint (*n* = 57)				
Class 5	1	56	2%	79%
Not in class	43	210	(0%–12%)	(74%–83%)
5				
	**Spontaneous resolution within 1 year (n)**	**No spontaneous resolution within 1 year (n)**	**Sensitivity (95% CI)**	**Specificity (95% CI)**
Class 1 – Polyarthritis (*n* = 62)				
Class 1	21	41	14%	74%
Not in class	130	118	(9%–20%)	(67%–80%)
1				
Class 2 – Oligoarthritis (*n* = 133)				
Class 2	56	77	37%	52%
Not in class	95	82	(30%–45%)	(44%–59%)
2				
Class 3 – Monoarthritis wrist (*n* = 27)				
Class 3	16	11	11%	93%
Not in class	135	148	(7%–17%)	(88%–96%)
3				
Class 4 – Monoarthritis small joint (*n* = 31)				
Class 4	17	14	11%	91%
Not in class	134	145	(7%–17%)	(86%–95%)
4				
Class 5 – Monoarthritis large joint (*n* = 57)				
Class 5	41	16	27%	90%
Not in class	110	143	(21%–35%)	(84%–94%)
5				

For each class the number of patients in that class and the number of patients that either develops RA or spontaneous resolution is shown. RA, rheumatoid arthritis; CI, confidence interval.

## Data Availability

All data relevant to the study are included in the article or uploaded as supplementary information. Additional data are available upon reasonable request.
