# Supplementary material for: Disentangling heterogeneity in contemporary undifferentiated arthritis – A large cohort study using latent class analysis
Source: Semin Arthritis Rheum. Author manuscript; Available in PMC 2024 Apr 25. (PMC7615885; doi:10.1016/j.semarthrit.2023.152251)
Supplement: Supplemental file [file EMS195476-supplement-Supplemental_file.docx]

**Supplementary files – Disentangling heterogeneity in contemporary UA**

[S1 - Supplementary data 1: Explanation latent class analysis 2](#_Toc132374413)

[S2 - Supplementary figure 1: Selection of study population 4](#_Toc132374414)

[S3 - Supplementary table 1: Baseline characteristics for patients with known DMARD-use & known autoantibodies and patients without known DMARD-use or autoantibodies. 5](#_Toc132374415)

[S4 – Supplementary table 2: Statistical measures per class 6](#_Toc132374416)

[S5 – Supplementary table 3: Baseline characteristics per class 7](#_Toc132374417)

[Supplementary References 8](#_Toc132374418)

S1 - Supplementary data 1: Explanation latent class analysis

Commonly used statistical methods such as regression analysis focus on relationships among variables and the goal is to predict outcomes. LCA on the other hand, is a person-centred approach: the goal is to group individuals that are similar and different from others.[1] These subgroups are called ‘latent classes’ because these subgroups are part of a larger population in which patients share certain characteristics, while these subgroups were possibly not evident (or unobserved) before the LCA. The subgroups are not easily distinguished based on, for example, a clinical diagnosis. The LCA uses categorical variables to detect mutually exclusive subgroups. In other words: LCA can detect latent heterogeneity in a population based on categorical patient characteristics.[2] LCA increases the number of classes stepwise, until the best model is identified.

Both statistical measures and clinical relevance are used to select the best fit model. Statistical measures are evaluated per model and compared with the model with one class less (i.e.: 3-class model is compared with the 2-class model). We used multiple statistical measures to select the statistically best-fit model.[2,3]

Since the commonly used log likelihood difference test cannot be used to test the nested latent class models, we used the Lo-Mendell-Rubin adjusted likelihood ratio test. This test compares the improvement in fit between the current model and the model with one class less. A p-value of <0.05 means that the current model is a statistically significant improvement in the fit by creating one additional class.

Statistical information criteria (IC) were used as well to guide the decision on the best-fit number of classes. Therefore the adjusted Bayesian Information Criterion (aBIC), Bayesian Information Criterion (BIC) and Akaike’s Information Criterion (AIC) were used. A lower IC indicates a better model fit.

Finally the entropy was assessed. Entropy ranges from 0-1 and indicates how accurately the model defines the classes. An entropy close to 1 is ideal. In other words: a higher value indicates a more precise assignment of an individual patient in that specific latent class.

After selecting the best fit model two things can be obtained: 1) a probability of class membership and 2) the probability of the observed variable (in our case the clinical characteristics) within each class. This means that the exact percentage of patients within each class or percentage of patients with a certain clinical characteristic cannot be determined based on LCA alone. For the best-fit model the probability of each clinical feature within the classes and across classes should be evaluated. Thereafter each class can be labelled by its clinically most recognizable features. Features can be dominant/have a high probability either within a class (differentiating within one class) or across a class (differentiating between classes).

S2 - Supplementary figure 1: Selection of study population


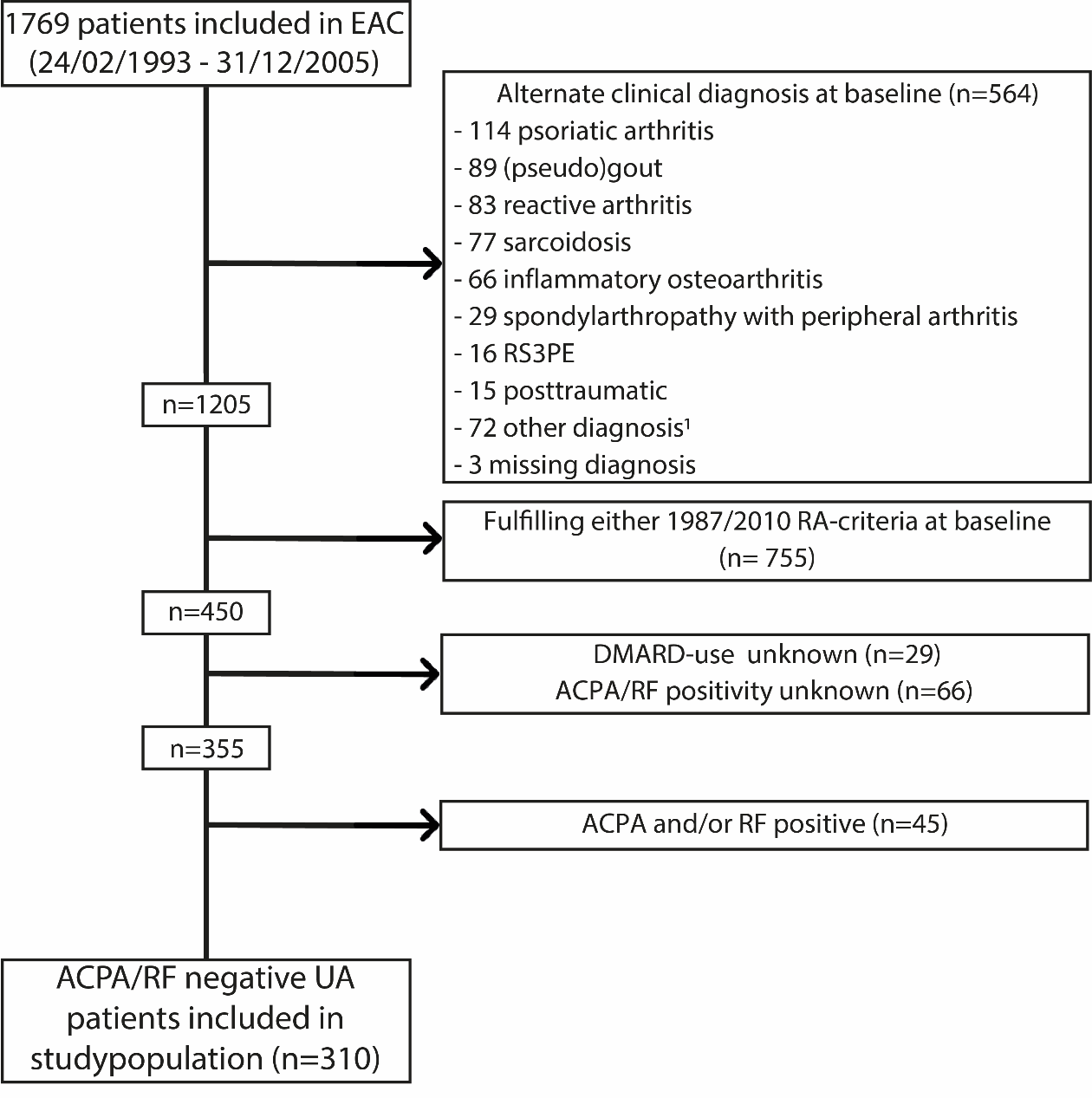
Legend: An overview of patients selected within the EAC cohort. Patients with unknown DMARD-use did not significantly differ from the studypopulation with known DMARD-use. Patients with unknown APCA and/or RF did not significantly differ from the population with known ACPA and/or RF, except for age and family history with RA. 1: other diagnosis: consists of multiple groups, including paramalignant (n=14), septic arthritis (n=10), lyme disease (n=8), systemic lupus erythematosus (n=8), juvenile chronic arthritis (n=4), mixed connective tissue disease/vasculitis (n=1), other (systemic) disease (n=27).

|  | **DMARD use & Autoantibody status known**  **n=355** | **DMARD use or Autoantibody status unknown**  **n=95** | **p-value** |
| --- | --- | --- | --- |
| Age at inclusion (years) | 49 (17) | 46 (18) | 0.086 |
| Male sex | 165 (46) | 42 (44) | 0.694 |
| Morning stiffness ≥60min | 82 (23) | 25 (26) | 0.520 |
| Symptom duration (days) | 93 (35-214) | 66 (19-195) | 0.155 |
| Subacute onset (<1 week) | 218 (61) | 54 (57) | 0.496 |
| Family history positive for RA | 57 (16) | 24 (25) | 0.047 |
| Swollen joint count (68-joints) | 2 (1-4) | 2 (1-3) | 0.319 |
| Monoarthritis | 134 (38%) | 34 (36%) | 0.814 |
| Oligoarthritis | 118 (33%) | 30 (32%) | 0.823 |
| Polyarthritis | 62 (17%) | 13 (14%) | 0.568 |
| Symmetry of swollen joints | 93 (26) | 22 (23) | 0.697 |
| Elevated CRP | 142 (40) | 35 (37) | 0.386 |
| Elevated ESR | 152 (43) | 37 (39) | 0.519 |
| Autoantibody positivity (RF or ACPA) | 45 (13) | 2 (8)^#^ | 0.456 |

S3 - Supplementary table 1: Baseline characteristics for patients with known DMARD-use & known autoantibodies and patients without known DMARD-use or autoantibodies.

Legend: Data are n (%), mean (SD) or median (IQR). Onset of symptoms was considered subacute in case the symptom duration was <1 week. CRP was considered elevated if ≥10mg/L, ESR was considered elevated depending on age and gender (<50 years: Male >15 mm/h, Female >20mm/h; >50 years: male >20mm/h, female>30mm/h considered elevated). ^#^could not be assessed in patients with unknown autoantibodies, therefore only assessed within patients with unknown DMARD-use.

S4 – Supplementary table 2: Statistical measures per class

|  | **Model log-likelihood value** | **LR test** | **BIC** | **aBIC** | **AIC** | **Entropy** | **Degrees of freedom** |
| --- | --- | --- | --- | --- | --- | --- | --- |
| 2-class | -2001.368 | 0.0000 | 4169.096 | 4077.119 | 4060.735 | 0.933 | 29 |
| 3-class | -1880.471 | 0.0000 | 4013.351 | 3873.799 | 3848.942 | 0.945 | 44 |
| 4-class | -1833.471 | 0.0000 | 4005.400 | 3818.275 | 3784.943 | 0.957 | 59 |
| **5-class** | **-1802.721** | **0.0002** | **4029.949** | **3795.249** | **3753.442** | **0.965** | **74** |
| 6-class | -1788.139 | 0.0534 | 4086.834 | 3804.559 | 3754.279 | 0.954 | 89 |

The best fit model (statistically and clinically most relevant) is presented in bold. In this case the 5-class model was the best fit. AIC, Akaike Information Criterion; BIC, Bayesian Information Criterion; aBIC, sample-size adjusted BIC; LR, likelihood ratio.

S5 – Supplementary table 3: Baseline characteristics per class

|  | Class 1  ‘polyarthritis’ | Class 2  ‘oligoarthritis’ | Class 3  ‘monoarthritis wrist’ | Class 4  ‘monoarthritis other small joint’ | Class 5  ‘monoarthritis large joint’ |
| --- | --- | --- | --- | --- | --- |
| Age at inclusion (years) | 52 (18) | 49 (16) | 53 (15) | 44 (15) | 46 (17) |
| Age >50 years | 31 (50) | 72 (54) | 15 (56) | 9 (29) | 23 (40) |
| Male sex | 25 (40) | 66 (50) | 14 (52) | 9 (29) | 34 (60) |
| BMI>25 | 12 (43) | 34 (47) | 8 (73) | 3 (19) | 12 (43) |
| MS ≥60 minutes | 12 (21) | 39 (32) | 2 (7) | 6 (20) | 10 (18) |
| Relative with RA | 8 (14) | 17 (14) | 9 (35) | 5 (17) | 7 (13) |
| Subacute onset (<1wk) | 33 (54) | 81 (65) | 20 (74) | 19 (61) | 42 (76) |
| Swollen joint count (68-joints) | 6 (5-8) | 2 (2-3) | 1 (1-1) | 1 (1-1) | 1 (1-1) |
| Monoarthritis present | 0 (0) | 0 (0) | 27 (100) | 31 (100) | 57 (100) |
| Monoarthritis MCP1-5/MTP2-5 | 0 (0) | 0 (0) | 0 (0) | 13 (42) | 0 (0) |
| Monoarthritis DIP/PIP | 0 (0) | 0 (0) | 0 (0) | 13 (42) | 0 (0) |
| Monoarthritis wrist | 0 (0) | 0 (0) | 27 (100) | 0 (0) | 0 (0) |
| Monoarthritis large joint | 0 (0) | 0 (0) | 0 (0) | 0 (0) | 57 (100) |
| Oligoarthritis | 0 (0) | 113 (100) | 0 (0) | 0 (0) | 0 (0) |
| Polyarthritis | 62 (100) | 0 (0) | 0 (0) | 0 (0) | 0 (0) |
| Symmetric arthritis | 44 (71) | 48 (37) | 0 (0) | 0 (0) | 0 (0) |
| APR elevated | 36 (58) | 72 (54) | 13 (48) | 8 (26) | 32 (56) |

Legend: Data are n (%), mean (SD) or median (IQR). Onset of symptoms was considered subacute in case the symptom duration was <1 week. CRP was considered elevated if ≥10mg/L, ESR was considered elevated depending on age and gender (<50 years: Male >15 mm/h, Female >20mm/h; >50 years: male >20mm/h, female>30mm/h considered elevated). Not all patients from class 4 classify into the monoarthritis mcp1-5/mtp2-5 or monoarthritis pip/dip groups, these patients presented with monoarthritis from mtp1 (n=3) and arthritis from the first interphalangeal joint of the foot (n=1). BMI: body mass index; MS: morning stiffness; MCP: metacarpophalangeal joint; MTP: metatarsophalangeal joint; DIP: distal interphalangeal joint; PIP: proximal interphalangeal joint; APR: acute phase reactants, consisting of ESR and CRP.

Supplementary References

1. Muthén B, Muthén LK. Integrating Person-Centered and Variable-Centered Analyses: Growth Mixture Modeling With Latent Trajectory Classes. 2000;24(6):882-91. doi: <https://doi.org/10.1111/j.1530-0277.2000.tb02070.x>

2. Weller BE, Bowen NK, Faubert SJ. Latent Class Analysis: A Guide to Best Practice. *Journal of Black Psychology* 2020;46(4):287-311. doi: 10.1177/0095798420930932

3. Nylund KL, Asparouhov T, Muthén BO. Deciding on the Number of Classes in Latent Class Analysis and Growth Mixture Modeling: A Monte Carlo Simulation Study. *Structural Equation Modeling: A Multidisciplinary Journal* 2007;14(4):535-69. doi: 10.1080/10705510701575396
